# Apoptosis-linked gene 2 promotes breast cancer growth and metastasis by regulating the cytoskeleton

**DOI:** 10.18632/oncotarget.13740

**Published:** 2016-12-01

**Authors:** Juan Qin, Dengwen Li, Yunqiang Zhou, Songbo Xie, Xin Du, Ziwei Hao, Ruming Liu, Xinqi Liu, Min Liu, Jun Zhou

**Affiliations:** ^1^ State Key Laboratory of Medicinal Chemical Biology, Key Laboratory of Bioactive Materials of the Ministry of Education, College of Life Sciences, Nankai University, Tianjin 300071, China; ^2^ Institute of Biomedical Sciences, College of Life Sciences, Key Laboratory of Animal Resistance Biology of Shandong Province, Shandong Normal University, Jinan 250014, China

**Keywords:** breast cancer, growth, metastasis, cell proliferation, cell migration

## Abstract

Breast cancer is the most prevalent cancer in women. Although it begins as local disease, breast cancer frequently metastasizes to the lymph nodes and distant organs. Therefore, novel therapeutic targets are needed for the management of this disease. Apoptosis-linked gene 2 (ALG-2) is a calcium-binding protein crucial for diverse physiological processes and has recently been implicated in cancer development. However, it remains unclear whether this protein is involved in the pathogenesis of breast cancer. Here, we demonstrate that the expression of ALG-2 is significantly upregulated in breast cancer tissues and is correlated with clinicopathological characteristics indicative of tumor malignancy. Our data further show that ALG-2 stimulates breast cancer growth and metastasis in mice. ALG-2 also promotes breast cancer cell proliferation, survival, and motility *in vitro*. Mechanistic data reveal that ALG-2 disrupts the localization of centrosome proteins, resulting in spindle multipolarity and chromosome missegregation. In addition, ALG-2 drives the polarization and migration of breast cancer cells by facilitating the rearrangement of microtubules and microfilaments. These findings reveal a critical role for ALG-2 in the pathogenesis of breast cancer and have important implications for its diagnosis and therapy.

## INTRODUCTION

Breast cancer is the most prevalent cancer among women, and efforts to improve its early detection and treatment are critically needed to improve the mortality rate. A number of biomarkers have been identified for use in breast cancer staging and personalization of therapeutic strategies [1]. However, metastasis of breast cancer to other tissues or organs dramatically worsens the prognosis and represents the greatest challenge to disease management [2]. Metastasis is a complicated multi-step process involving dissemination of tumor cells from the primary tumor, invasion of the matrix, entry into the circulatory system, extravasation through the capillary endothelium, and finally outgrowth of secondary tumors in distant organs [2]. At the molecular level, these processes require changes in cell polarity that are driven by substantial reorganization of the cytoskeleton [3]. However, our knowledge of the precise contributions of different cytoskeletal components and their respective regulators to metastasis is still fragmented and needs to be expanded in order to improve long-term therapeutic management of breast cancer.

Apoptosis-linked gene 2 (ALG-2) is a calcium-binding protein that contains five serially repeated EF-hand motifs, placing this protein in the penta-EF-hand family [4–6]. ALG-2 was originally described as a pro-apoptotic protein based on the results of a death-trap differential screen used to identify new apoptosis-related genes in T-cell hybridoma cells and was suggested to participate in T cell receptor-, Fas-, and glucocorticoid-induced apoptosis [7, 8]. However, apoptosis was not inhibited in ALG-2-deficient mice [9], suggesting that the protein is functionally redundant. The calcium-binding domain of ALG-2 plays a significant role in its homodimerization, driving structural changes required for binding to various intracellular protein partners [6]. ALG-2 is expressed in all tissues and cell lines analyzed to date and has been implicated in diverse physiological processes, including endoplasmic reticulum stress-induced cell death, neuronal apoptosis during organ formation, signal transduction, membrane trafficking, and post-transcriptional control of gene expression [6].

Recent studies suggest that changes in ALG-2 expression may contribute to cancer development. ALG-2 has been found to be upregulated in lung cancer and liver cancer tissues [10]. In contrast, lower ALG-2 mRNA expression was significantly correlated with poor overall survival in gastric cancer [11]. These findings suggest that the roles of ALG-2 may vary in different cancer types. At present, it is unknown whether ALG-2 is involved in the pathogenesis of breast cancer. In this study, we demonstrate that ALG-2 expression is markedly elevated in breast cancer tissues and is correlated with clinicopathological characteristics. Our data also show that ALG-2 has an important function in breast cancer growth and metastasis by regulating cytoskeletal components and stimulating cell proliferation and migration. These findings suggest a potential for ALG-2 in the diagnosis and therapy of breast cancer.

## RESULTS

### Upregulation of ALG-2 in breast cancer tissues is correlated with clinicopathological indicators of tumor malignancy

**Figure 1:**
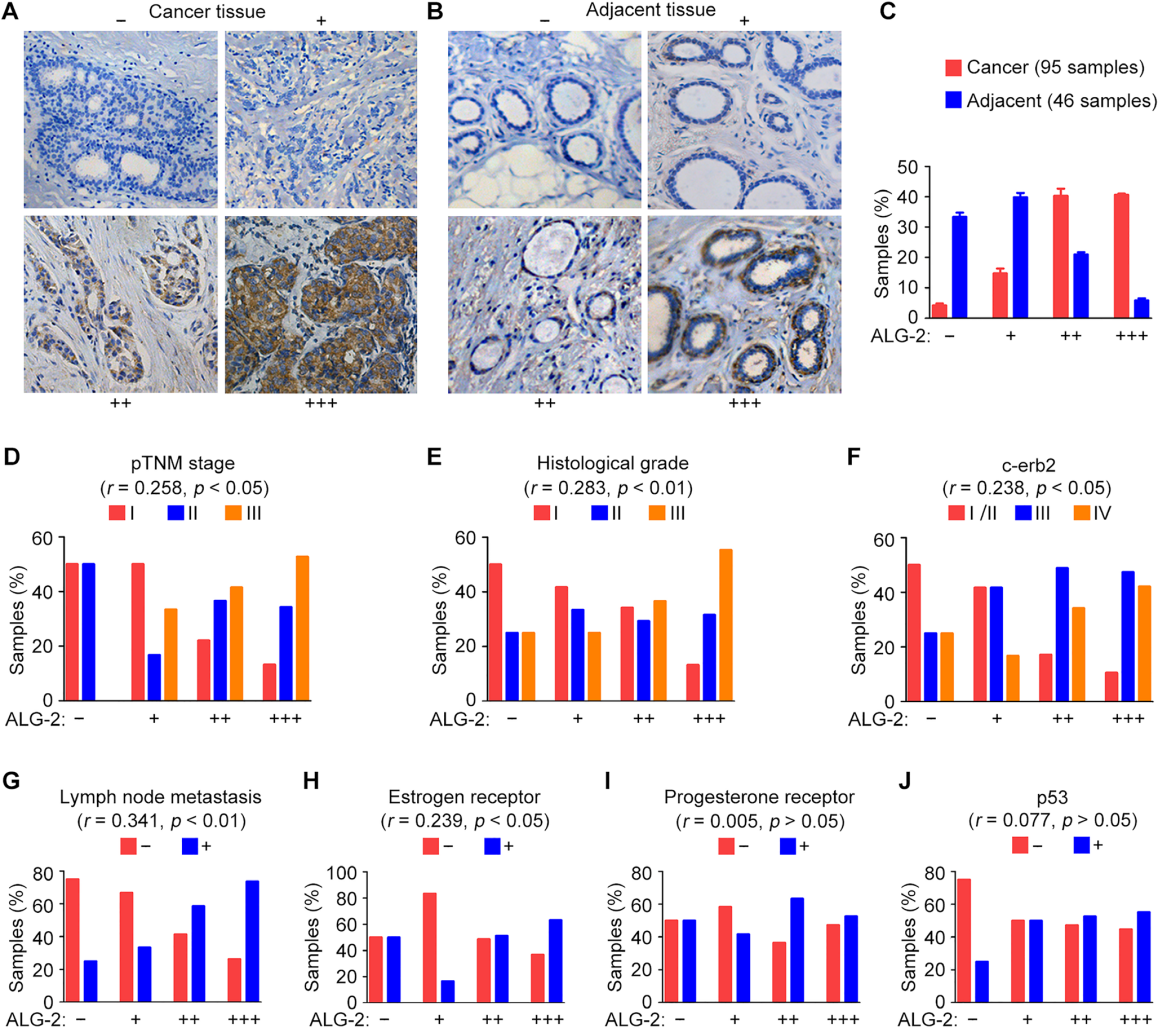
Upregulation of ALG-2 in breast cancer tissues is associated with clinicopathological parameters related to tumor malignancy (**A** and **B**) Representative images of immunohistochemical staining of ALG-2 expression in breast cancer tissues (A) and adjacent tissues (B). Samples were classified into four groups based on ALG-2 staining intensity and the percentage of stained cells. (**C**) Quantification of ALG-2 expression in 95 breast cancer tissue samples and 46 adjacent tissue samples. (**D**–**J**) Analysis of correlations between ALG-2 expression and clinicopathological parameters, including the pTNM stage (D), histological grade (E), c-erb2 expression (F), lymph node metastasis (G), estrogen receptor positivity (H), progesterone receptor positivity (I), and p53 positivity (J). Error bars indicate means ± SEM.

To investigate the association between ALG-2 expression and breast cancer, we performed immunohistochemical staining to analyze the expression of ALG-2 in samples from patients who underwent surgical resection. A total of 95 breast cancer tissue samples and 46 adjacent tissue samples were analyzed. ALG-2 was undetectable or marginally detectable in the majority of adjacent tissues, but strong expression of ALG-2 was observed predominantly in the cytoplasm of breast cancer cells (Figure 1A, 1B). The samples were classified into four groups based on staining intensity and the percentage of stained cells (Figure 1A, 1B). We found that 89.5% of breast cancer tissues, but only 26.1% of adjacent tissues, exhibited elevated expression of ALG-2 (++ and +++). Absence of ALG-2 expression (−) was only observed in 3.6% of breast cancer samples (Figure 1C). ALG-2 expression was low in ductal carcinoma *in situ*, whereas a significant increase in ALG-2 expression was observed in invasive breast carcinoma (Table 1).

**Table 1:** The correlation between ALG-2 expression and clinicopathological parameters

Clinicopathological parameters	ALG-2 staining									
		−		+		++		+++		*p* value
		*n*	%	*n*	%	*n*	%	*n*	%	
Age	< 40	0	0	1	8	2	5	3	8	*p* > 0.05
	40–60	1	25	4	33	16	39	15	39	
	> 60	3	75	7	58	23	56	20	53	
Tumor type	DCIS	4	100	10	83	9	22	5	13	*p* < 0.01
	IBC	0	0	2	17	32	78	33	87	
pTNM stage	I	2	50	6	50	9	22	5	13	*p* < 0.05
	II	2	50	2	17	15	37	13	34	
	III/IV	0	0	4	33	17	41	20	53	
Histological grade	I	2	50	5	42	14	34	5	13	*p* < 0.01
	II	1	25	4	33	12	29	12	32	
	III	1	25	3	25	15	37	21	55	
LNM	−	3	75	8	67	17	41	10	26	*p* < 0.01
	+	1	25	4	33	24	59	28	74	
C-erb2	I/II	2	50	5	42	7	17	4	11	*p* < 0.05
	III	1	25	5	42	20	49	18	47	
	IV	1	25	2	17	14	34	16	42	
ER	−	2	50	10	83	20	49	14	37	*p* < 0.05
	+	2	50	2	17	21	51	24	63	
PR	−	2	50	7	58	15	37	18	47	*p* > 0.05
	+	2	50	5	42	26	63	20	53	
p53	−	3	75	6	50	18	47	17	45	*p* > 0.05
	+	1	25	6	50	20	53	21	55	

Abbreviations: DCIS, ductal carcinoma *in situ*; IBC, invasive breast carcinoma; LNM, lymph node metastasis; ER, estrogen receptor; PR, progesterone receptor.

We next analyzed associations between ALG-2 upregulation and clinical features of breast cancer. Results from these analyses revealed that ALG-2 overexpression was significantly correlated with several variables associated with poor prognosis [12], such as higher pathological tumor node metastasis (pTNM) stage (Figure 1D), higher histological grade (Figure 1E), c-erb2 positivity (Figure 1F), higher incidence of lymph node metastasis (Figure 1G), and estrogen receptor positivity (Figure 1H). No significant correlation was identified between ALG-2 and progesterone receptor (Figure 1I), p53 (Figure 1J), or age (Table 1).

### ALG-2 stimulates the growth and metastasis of breast cancer in a rodent xenograft model

**Figure 2:**
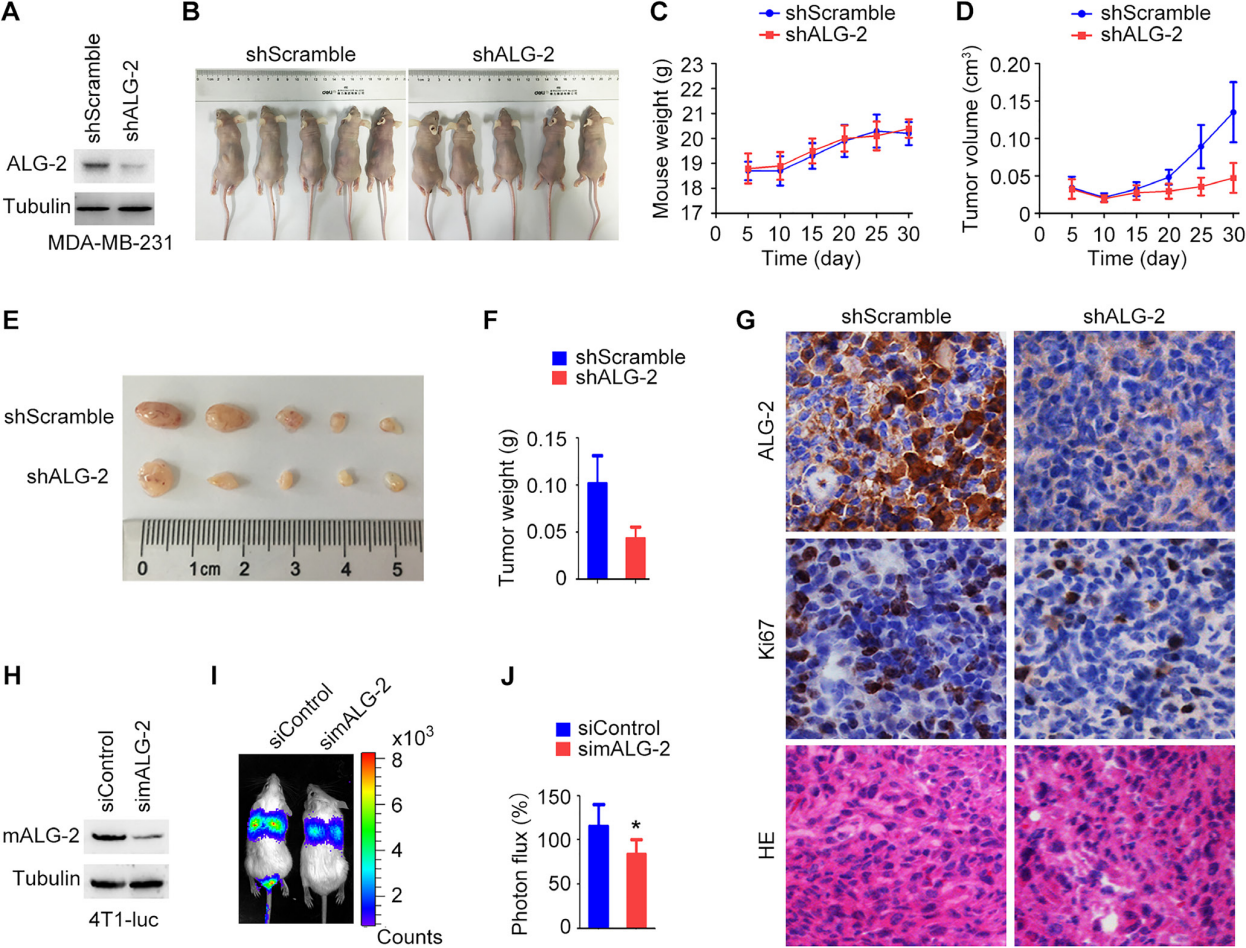
ALG-2 stimulates breast cancer growth and metastasis in mice (**A**) Immunoblot analysis of ALG-2 and α-tubulin in MDA-MB-231/shALG-2 and MDA-MB-231/shScramble cells. (**B**) MDA-MB-231/shALG-2 or MDA-MB-231/shScramble cells were injected subcutaneously into athymic nude mice, and photographs were taken 30 days later. (**C** and **D**) Mouse weight (C) and tumor volume (D) were measured every 5 days. (**E** and **F**) Tumors were isolated from mice sacrificed 30 days post-injection. Tumors were photographed (E), and tumor weight was measured (F). (**G**) Immunohistochemical staining of ALG-2 and Ki67 expression and HE staining of xenograft tumor sections. (**H**) Immunoblot analysis of mALG-2 and α-tubulin in 4T1-luc cells transfected with control or mALG-2 siRNAs. (**I**) 4T1-luc cells transfected with control or mALG-2 siRNAs were injected into BALB/c mice (5 per group). Luciferin was injected intraperitoneally to detect lung metastasis by bioluminescence imaging 6 days later. (**J**) Analysis of the luminescence photon flux. Error bars indicate means ± SEM. *p < 0.05.

Because ALG-2 overexpression was correlated with characteristics associated with poor prognosis of breast cancer patients, we next analyzed the effects of ALG-2 knockdown *in vivo* using a mouse xenograft model. MDA-MB-231/shALG-2 cells, which had stable ALG-2 knockdown (Figure 2A), were injected subcutaneously into athymic nude mice (Figure 2B). Analysis of mouse weight revealed no significant differences between the MDA-MB-231/shALG-2 and MDA-MB-231/shScramble groups (Figure 2C). However, the volumes and weights of tumors in the MDA-MB-231/shALG-2 group were substantially decreased relative to the control group (Figure 2D–2F). Immunohistochemical analysis of the xenograft tumor tissues revealed that the percentage of Ki67-positive cells was lower in the MDA-MB-231/shALG-2 group (Figure 2G). In addition, HE staining showed that the percentage of cells with apoptotic nuclear morphology was higher in the MDA-MB-231/shALG-2 group (Figure 2G).

We also investigated whether ALG-2 plays a role in breast cancer metastasis by injecting 4T1-luc cells, mouse breast cancer cells expressing luciferase, into the tail veins of BALB/c mice. Transfection of mALG-2-specific siRNAs effectively reduced the expression of mALG-2 in 4T1-luc cells (Figure 2H). Intraperitoneal injection of luciferin was then used to detect lung metastases via bioluminescence imaging. Relative levels of metastasis were quantified by measuring the luminescence photon flux 7 days after injection (Figure 2I, 2J). We found that knockdown of mALG-2 expression significantly inhibited metastasis of 4T1-luc cells to the lung (Figure 2I, 2J). Taken together, these data indicate that ALG-2 depletion inhibits breast cancer growth and metastasis *in vivo*.

### ALG-2 promotes the proliferation and survival of breast cancer cells

**Figure 3:**
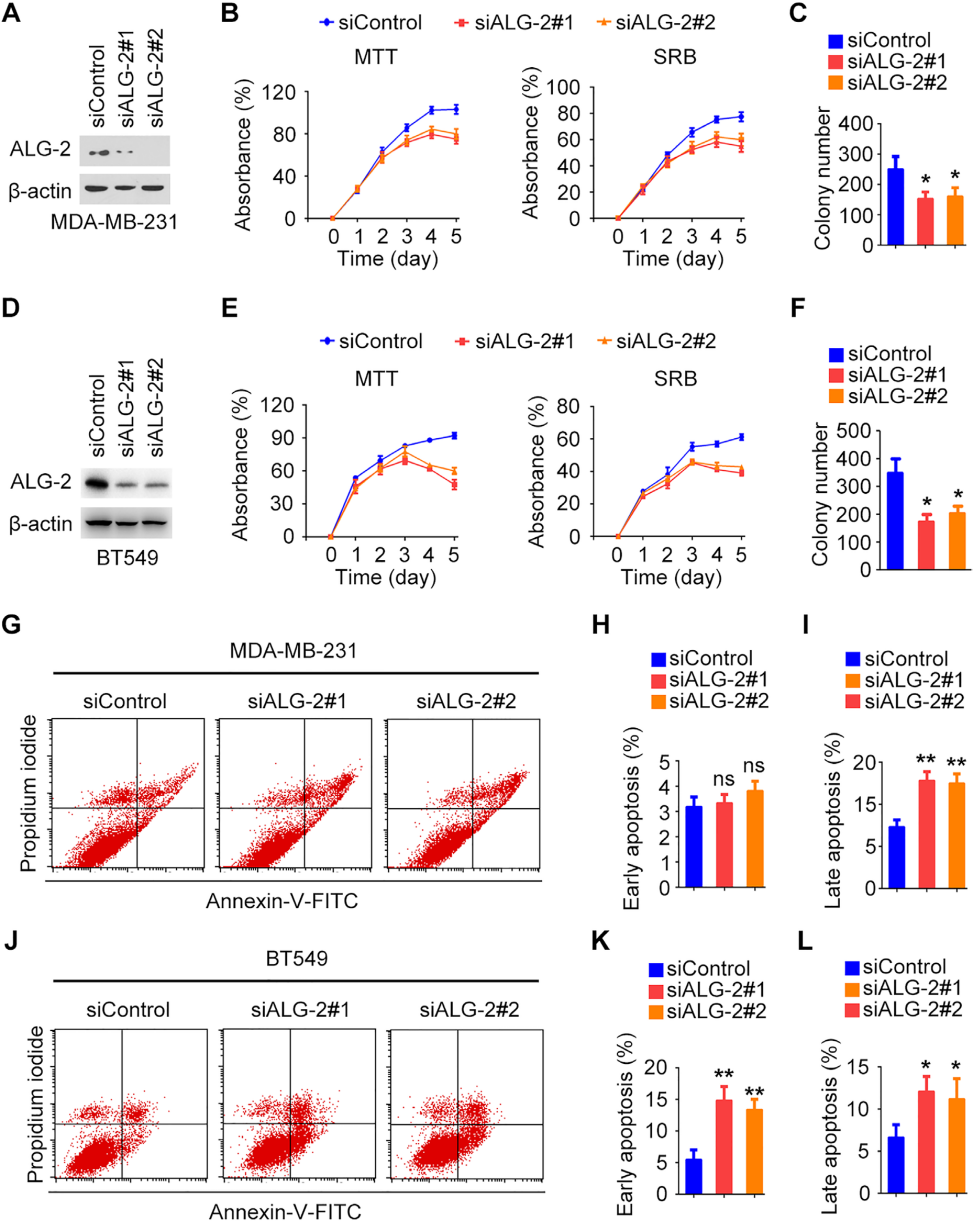
ALG-2 promotes the proliferation and survival of breast cancer cells (**A** and **D**) Immunoblot analysis of ALG-2 and β-actin in MDA-MB-231 (A) and BT549 (D) cells. (**B** and **E**) Silencing of ALG-2 inhibits the proliferation of MDA-MB-231 (B) and BT549 (E) cells as determined by MTT and SRB assays. (**C** and **F**) Colony formation by control or ALG-2 siRNA-transfected MDA-MB-231 (C) and BT549 (F) cells cultured for 2 weeks. (**G** and **J**) Flow cytometric analysis of apoptosis in MDA-MB-231 (G) and BT549 (J) cells 72 hours after transfection with control or ALG-2 siRNAs. (**H** and **I**) Experiments were performed as in G, and the percentages of early apoptotic cells (annexin V-FITC+, propidium iodide-) and late apoptotic cells (annexin V-FITC+, propidium iodide+) were quantified. For each group, 1 × 10^5^ cells were counted. (**K** and **L**) Experiments were performed as in J, and the percentages of early and late apoptotic cells were quantified. For each group, 1 × 10^5^ cells were counted. Error bars indicate means ± SEM. *p < 0.05; **p < 0.01; ns, not significant.

To gain insight into the functions of ALG-2 in the pathogenesis of breast cancer, we analyzed its effects on cell proliferation and survival *in vitro*. Expression of endogenous ALG-2 was effectively knocked down by transfection with ALG-2 siRNAs in both MDA-MB-231 and BT549 cells (Figure 3A, 3D). Sulforhodamine B (SRB) and 3-(4,5-dimethylthiazolyl-2)-2,5-diphenyltetrazolium bromide (MTT) assays revealed that depletion of ALG-2 resulted in significant inhibition of the proliferation and survival of MDA-MB-231 and BT549 cells (Figure 3B, 3E). To assess whether ALG-2 depletion affected colony formation, siRNA-transfected cells were cultured for 2 weeks, and the resulting colonies were stained with crystal violet and counted. Knockdown of ALG-2 expression significantly decreased the colony-forming efficiency of MDA-MB-231 and BT549 cells (Figure 3C, 3F). These results are consistent with the *in vivo* results demonstrating that downregulation of ALG-2 results in inhibition of tumor growth.

Flow cytometry was then performed to assess the levels of apoptosis at 72 hours after transfection of cells with ALG-2 siRNAs in MDA-MB-231 cells and BT549 cells (Figure 3G, 3J). In MDA-MB-231 cells, the percentage of late apoptotic cells (annexin V-FITC^+^, propidium iodide^+^) was significantly increased by transfection with ALG-2 siRNAs (Figure 3I), whereas the percentage of early apoptotic cells (annexin V-FITC^+^, propidium iodide^−^) was not obviously affected (Figure 3H). In contrast, in BT549 cells, the percentages of early and late apoptotic cells were both increased in the ALG-2 siRNA-treated group in comparison to the control group (Figure 3K, 3L). These results suggest that ALG-2 promotes cell survival by inhibiting apoptosis.

### Ectopic expression of ALG-2 triggers spindle multipolarity and chromosome missegregation

**Figure 4:**
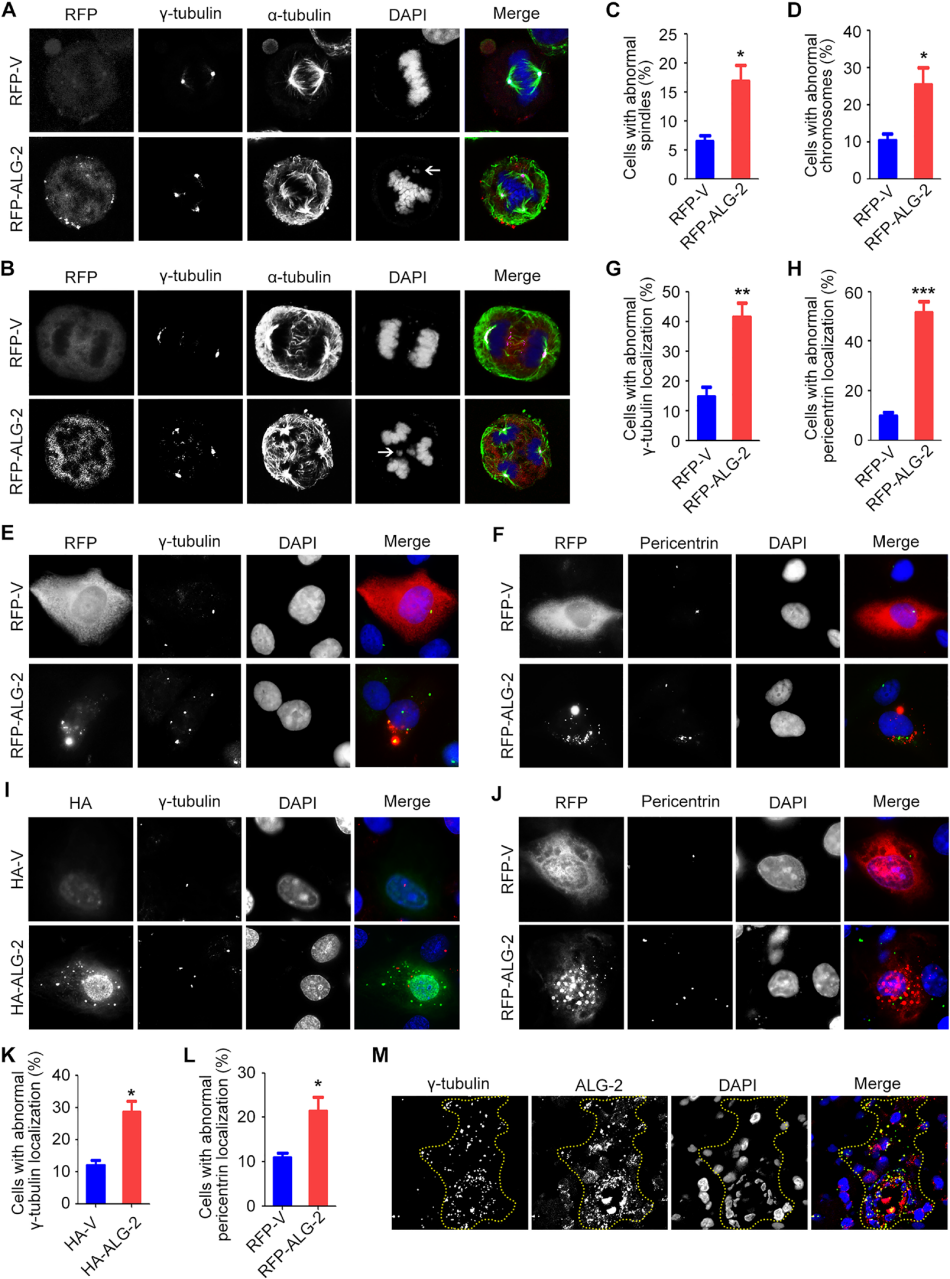
Ectopic expression of ALG-2 triggers spindle multipolarity and chromosome missegregation (**A** and **B**) MDA-MB-231 cells were transfected with RFP-ALG-2 or empty RFP vector (RFP-V) and stained with anti-γ-tubulin and anti-α-tubulin antibodies and DAPI. Arrows indicate unattached (A) or missegregated (B) chromosomes. (**C** and **D**) Experiments were performed as in A and B, and the percentages of cells with abnormal spindles (C) and abnormal chromosomes (D) were quantified. For each group, 100 mitotic cells were quantified. (**E** and **F**) MDA-MB-231 cells were transfected with RFP-ALG-2 or RFP-V and stained with anti-γ-tubulin (E) or anti-pericentrin antibodies (F) and DAPI. (**G** and **H**) Experiments were performed as in E and F, and the percentage of cells with abnormal localization of γ-tubulin (G) or pericentrin (H) was quantified. For each group, 300 cells were quantified. (**I** and **J**) MCF-10A cells were transfected with HA-ALG-2 or empty HA vector (HA-V) and stained with anti-γ-tubulin (I) or anti-pericentrin (J) antibodies and DAPI. (**K** and **L**) Experiments were performed as in I and J, and the percentage of cells with abnormal localization of γ-tubulin (K) or pericentrin (L) was quantified. For each group, 100 cells were quantified. (**M**) Immunofluorescence images of human breast cancer tissues stained with anti-γ-tubulin and anti-ALG-2 antibodies and DAPI. Dashed lines indicate cells with high ALG-2 expression. Error bars indicate means ± SEM. *p < 0.05; **p < 0.01; ***p < 0.001.

To understand the molecular mechanisms by which elevated expression of ALG-2 promotes breast tumorigenesis, we transfected MDA-MB-231 cells with an RFP-ALG-2 overexpression plasmid and assessed mitotic cells by immunostaining of microtubules and the spindle pole/centrosome marker γ-tubulin (Figure 4A, 4B). We found that the percentage of cells with multipolar spindles was significantly increased upon ALG-2 overexpression (Figure 4A–4C). Consistent with the spindle defects, unattached or missegregated chromosomes were more frequently detected in cells transfected with ALG-2 (Figure 4A, 4B, 4D).

We then examined whether the formation of multipolar spindles results from centrosome defects, by analyzing the localization of centrosome proteins in interphase cells. In cells transfected with the empty vector, we observed typical perinuclear centrosome localization of γ-tubulin (Figure 4E). In contrast, γ-tubulin was present in multiple irregular aggregates that colocalized with ectopic ALG-2 in a significant amount of cells overexpressing RFP-ALG-2, indicative of the presence of abnormal centrosomes (Figure 4E, 4G). Similar results were observed by analyzing the localization of pericentrin, another centrosome marker (Figure 4F, 4H). In addition, similar results were found in MCF-10A immortalized human breast epithelial cells (Figure 4I–4L).

To validate these results, we examined the localization of γ-tubulin in human breast cancer tissues by immunofluorescence microscopy. Consistent with the data obtained in MDA-MB-231 and MCF-10A cells, abnormal localization of γ-tubulin was frequently observed in cells with high ALG-2 expression (Figure 4M). Collectively, these data suggest that ectopic expression of ALG-2 results in centrosome abnormalities, leading to chromosome instability and potentially tumorigenesis.

### ALG-2 is important for the motility of breast cancer cells

**Figure 5:**
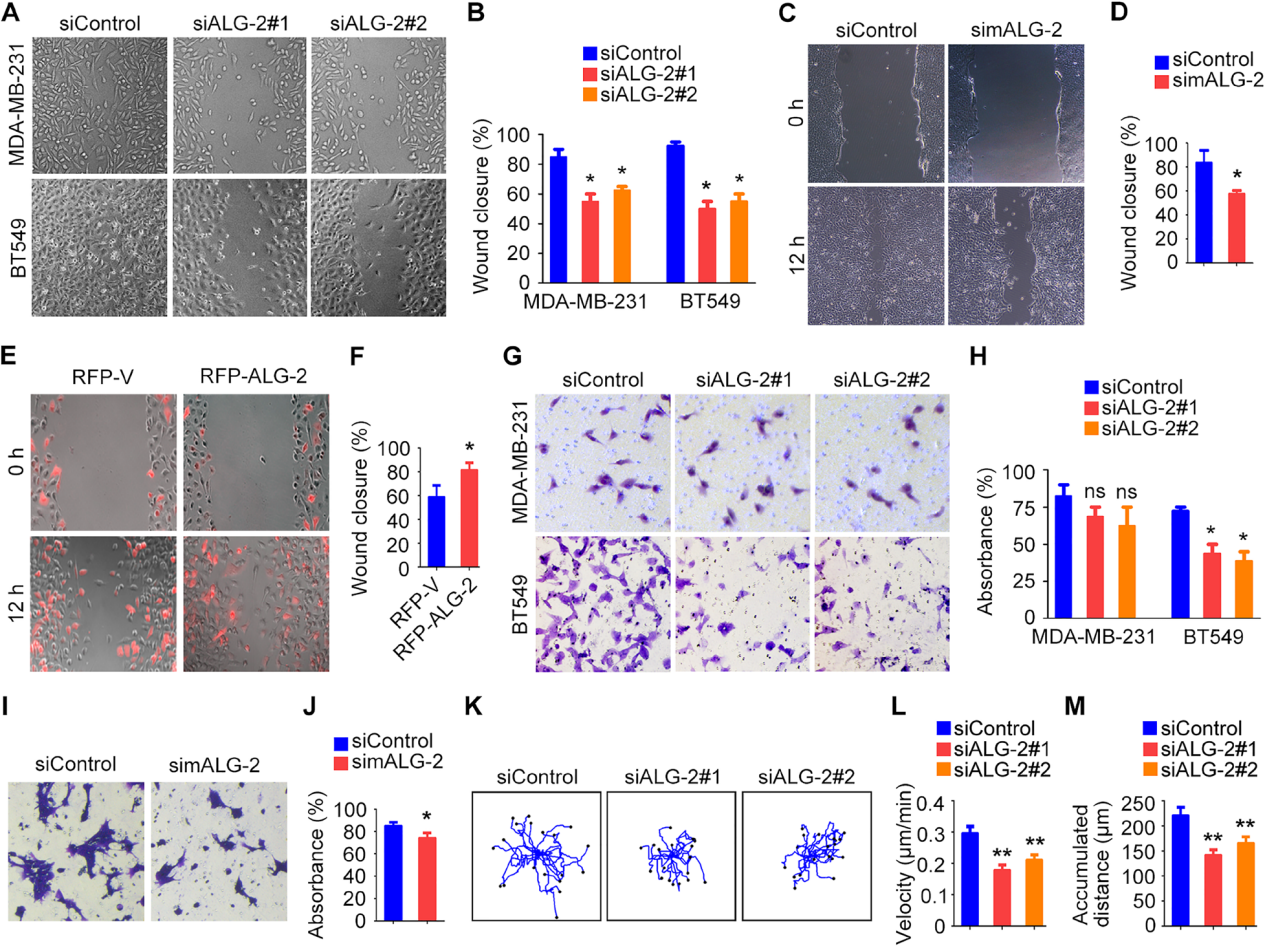
ALG-2 is important for the motility of breast cancer cells (**A**) Wound healing assays using MDA-MB-231 and BT549 cells transfected with control or ALG-2 siRNAs. Wound margins were imaged 16 hours after wounding. (**B**) Experiments were performed as in A, and the percentage of wound closure was quantified. (**C**) Wound healing assays using 4T1-luc cells transfected with control or mALG-2 siRNAs. (**D**) Experiments were performed as in C, and the percentage of wound closure was quantified. (**E**) Wound healing assays using BT549 cells transfected with RFP-V or RFP-ALG-2. (**F**) Experiments were performed as in E, and the percentage of wound closure was quantified. (**G**) Transwell migration assays using MDA-MB-231 and BT549 cells transfected with control or ALG-2 siRNAs. (**H**) Experiments were performed as in G, and the extent of transwell migration was quantified. (**I**) Transwell migration assays using 4T1-luc cells transfected with control or mALG-2 siRNAs. (**J**) Experiments were performed as in I, and the extent of transwell migration was quantified. (**K**–**M**) Analysis of random migration of MDA-MB-231 cells. Cell movement paths were tracked (K), and the velocity of cell movement (L) and the accumulated distance were analyzed (M). Error bars indicate means ± SEM. *p < 0.05; **p < 0.01; ns, not significant.

Because abnormal cell motility is a requirement for cancer metastasis [3], we performed wound healing and transwell assays to assess the role of ALG-2 in the motility of breast cancer cells. Wound healing assays were performed by scratching monolayers of MDA-MB-231 and BT549 cells transfected with control or ALG-2 siRNAs. We found that ALG-2 siRNAs inhibited wound closure, with less migrating cells filling in the wound region for both cell types (Figure 5A, 5B). Similar results were observed in 4T1-luc cells transfected with mALG-2 siRNAs (Figure 5C, 5D). In addition, wound healing assays revealed that overexpression of RFP-ALG-2 promoted the migration of BT549 cells (Figure 5E, 5F). Transwell assays further showed that ALG-2 depletion compromised the invasion of BT549 and 4T1-luc cells into the bottom surface of the transwell chambers, but only had subtle effect on MDA-MB-231 cells (Figure 5G–5J).

To gain additional insight into the effects of ALG-2 on cell motility, we examined random migration of single cells using time-lapse microscopy. We captured images every 5 minutes over a span of 12 hours and quantified the velocity of cell migration and the distance migrated using the Image J software (Figure 5K). ALG-2 depletion reduced the velocity of cell migration (Figure 5L) and decreased the accumulated distance of random cell movement (Figure 5M). Taken together, these data indicate that ALG-2 is critical for breast cancer cell motility.

### ALG-2 regulates cytoskeletal rearrangement in migrating breast cancer cells

**Figure 6:**
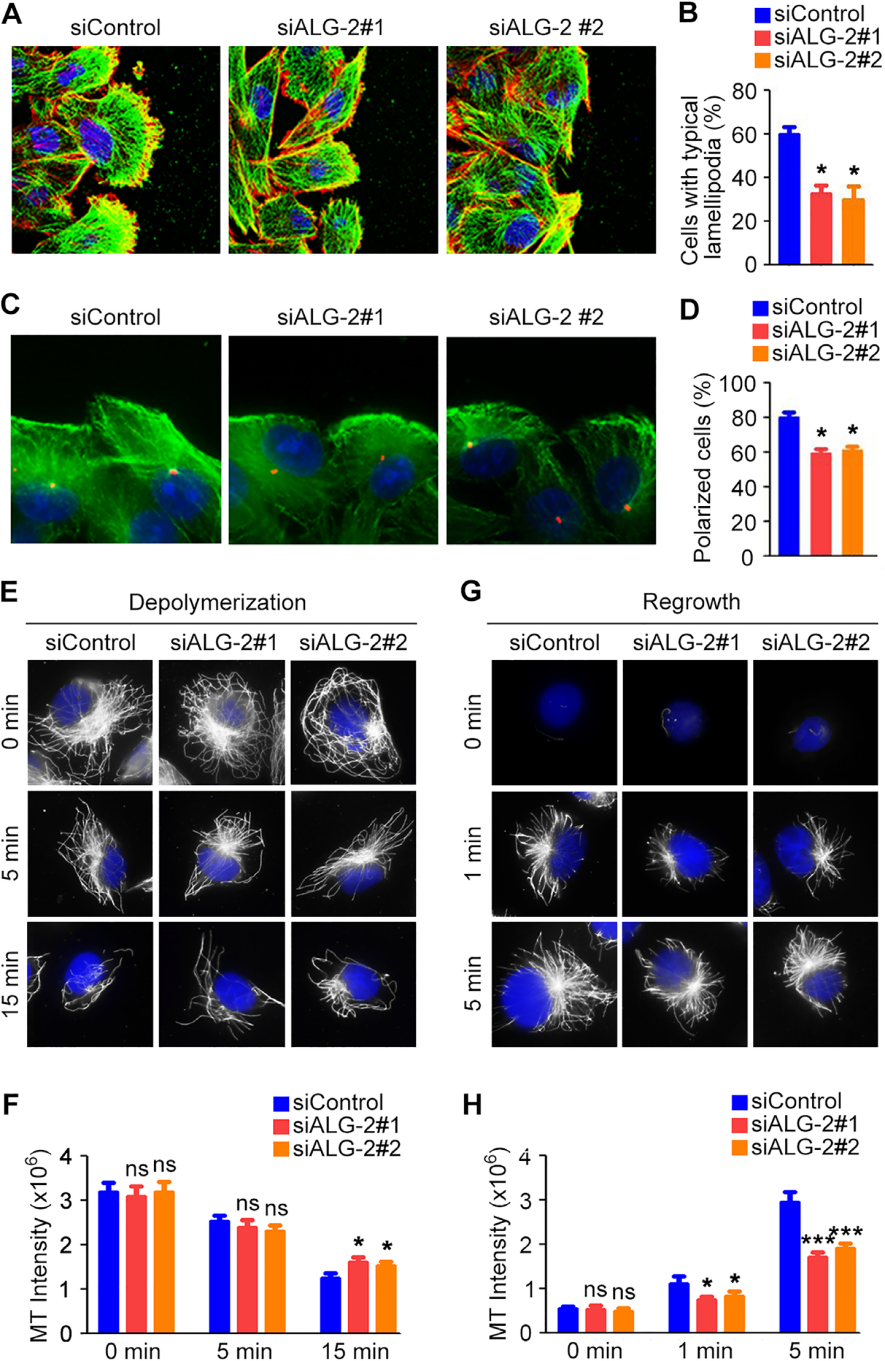
ALG-2 regulates cytoskeletal rearrangement in migrating breast cancer cells (**A**) MDA-MB-231 cells were transfected with control or ALG-2 siRNAs, and wound healing assays were performed. Cells were stained 3 hours after wounding with phalloidin (red), anti-α-tubulin antibody (green), and DAPI (blue). (**B**) Experiments were performed as in A, and the percentage of cells with typical lamellipodia was quantified. (**C**) Cells transfected with control or ALG-2 siRNAs were stained 3 hours after wounding with anti-γ-tubulin (red) and anti-α-tubulin (green) antibodies and DAPI (blue). (**D**) Experiments were performed as in C, and the percentage of cells at the wound margin with centrosomes localized in the forward-facing one-third of the cell was analyzed. (**E**–**H**) MDA-MB-231 cells transfected with control or ALG-2 siRNAs for 48 hours were incubated on ice to depolymerize microtubules. The intensity of the remaining microtubules was examined by staining with anti-α-tubulin antibody (E) and quantified using the Image J software (F). After complete microtubule depolymerization, cells were placed back at 37°C for the indicated time, and the intensity of microtubule regrowth was examined by staining with anti-α-tubulin antibody (G) and quantified using Image J (H). Error bars indicate means ± SEM. *p < 0.05; ***p < 0.001; ns, not significant.

Directed cell migration during wound healing requires the polarization of cells at the wound margin [13]. To assess whether the observed differences in cell motility were coupled with concomitant differences in cell polarization, we quantified changes in cellular morphology by analyzing the cytoskeleton. MDA-MB-231 cell monolayers transfected with control or ALG-2 siRNAs were scratched, fixed 3 hours later, and stained with phalloidin to visualize microfilaments and α-tubulin antibody to visualize microtubules. In the control group, cells at the wound margin exhibited a typical polarized morphology, with a microfilament-rich lamellipodium extension (Figure 6A, 6B). In contrast, the percentage of cells with typical lamellipodia were significantly decreased in the ALG-2 siRNA-treated groups (Figure 6A, 6B).

To further assess the polarized morphology during cell movement, wounded MDA-MB-231 monolayers were stained with γ-tubulin antibody to visualize the centrosome. The percentage of cells at the wound margin with centrosomes present in the leading third of the cell was quantified. In the control group, cells at the wound margin exhibited a typical polarized structure, with the centrosome positioned between the nucleus and the leading edge in approximately 80% of cells (Figure 6C, 6D). In contrast, knockdown of ALG-2 significantly inhibited reorientation of the centrosome in a polarized manner, evident as a significant decrease in the number of cells with centrosomes in the leading third of the cell (Figure 6C, 6D).

Microtubule stabilization at the leading edge has been shown to play a critical role in forward protrusion in migrating cells [13]. Thus, we next investigated whether ALG-2 plays a regulatory role in microtubule reorganization, which is critical for cell polarization and migration. Knockdown of ALG-2 had a modest effect on microtubule stability when cells were placed on ice to depolymerize microtubules (Figure 6E, 6F). ALG-2 also appeared to promote microtubule regrowth after depolymerization, as the intensity of microtubule staining was decreased in ALG-2 knockdown groups (Figure 6G, 6H). Together, these findings suggest that ALG-2 plays a role in the regulation of microtubule rearrangement in migrating breast cancer cells.

## DISCUSSION

During development, cell fate is tightly controlled by specific genes, and inappropriate expression or regulation of these genes can result in a variety of diseases [14]. Previous findings demonstrating that ALG-2 is dysregulated in several cancer types led us to investigate its role in breast cancer. Our results reveal that ALG-2 is markedly upregulated in breast cancer tissues and is associated with various clinicopathological parameters indicative of tumor malignancy. Our data also show that reduced ALG-2 expression interferes with the proliferative and metastatic potential of human breast cancer cells in a rodent xenograft model and *in vitro*. These findings suggest a critical role for ALG-2 in the pathogenesis of breast cancer and have important implications for the diagnosis and therapy of this disease.

The roles of ALG-2 in cell survival and apoptosis have been controversial. ALG-2 was initially reported to function as a pro-apoptotic protein that protects mouse T cell hybridoma cells from death induced by T cell receptor stimulation [7, 8], but this initial finding could not be confirmed by studies in T cells isolated from ALG-2 knockout mice [9]. High expression of ALG-2 has been reported in liver and lung cancers [10], suggesting that the protein plays a role in survival pathways. In addition, ALG-2 has been implicated in ovarian cancer progression, and it may be an independent predictor of progression-free survival [15]. These studies, together with our finding that ALG-2 is overexpressed in breast cancer, suggest that the protein plays a role in cell survival under certain circumstances.

Centrosome aberrations, including both structural and functional defects, have been strongly implicated in tumorigenesis [16]. Our results demonstrate that ALG-2 overexpression in breast cancer cells disrupts the localization of γ-tubulin and pericentrin and results in the formation of centrosome protein aggregates. However, how the mislocalization of centrosome components leads to tumorigenesis remain to be elucidated. Given that spindle multipolarity and chromosome missegregation are evident in cells overexpressing ALG-2, it is possible that the disruption of centrosome proteins by ALG-2 triggers chromosome instability, which may further contribute to tumorigenesis.

Our study demonstrates that ALG-2 plays a role in the regulation of cytoskeletal rearrangement that drives cell polarization and migration in breast cancer cells. At present, the molecular mechanism for how ALG-2 modulates the cytoskeleton is unclear. It will be helpful to perform kinetic live studies of the cytoskeleton in the future to better understand the timing and mechanism of action of ALG-2. It has been reported that calcium binding induces conformational changes in ALG-2 that modulate interactions between ALG-2 and various proteins [6]. Many candidate proteins proposed to interact with ALG-2 have cytoskeleton-related functions, such as Aurora A and microtubule-associated protein 4 [17]. Aurora A regulates centrosome maturation, entry into mitosis, formation and function of the bipolar spindle, microtubule dynamics, and control of cell polarity [18]. Microtubule-associated protein 4 is a major non-neuronal microtubule-associated protein that regulates microtubule polymerization and cellular morphology [19]. Thus, it is tempting to speculate that ALG-2 may regulate cytoskeletal rearrangement through interactions with specific cytoskeleton-associated proteins.

## MATERIALS AND METHODS

### Ethics statement

Investigation has been conducted in accordance with the ethical standards according to the Declaration of Helsinki and the national and international guidelines, and has been approved by the authors’ institutional review board.

### Materials

Antibodies against ALG-2 (sc-292580, Santa Cruz Biotechnology for immunoblotting; ab56933, Abcam for immunofluorescence and immunohistochemistry), α-tubulin (ab18251, Abcam), β-actin (A5316, Sigma-Aldrich), γ-tubulin (T3320, Sigma-Aldrich), pericentrin (PRB-432C, Covance), and Ki67 (ab66155, Abcam) were purchased from the indicated sources. Horseradish peroxidase-conjugated secondary antibodies were from Santa Cruz Biotechnology. Fluorescein isothiocyanate (FITC)- and rhodamine-conjugated secondary antibodies were from Jackson ImmunoResearch Laboratories. Rhodamine-conjugated phalloidin was from Sigma-Aldrich. SRB, MTT, and 4',6-diamidino-2-phenylindole (DAPI) were from Songon Biotech.

### Plasmids, siRNAs, and transfections

The retroviral plasmid for shRNA-mediated knockdown of human ALG-2, pGFP-V-RS-shALG-2, was constructed by insertion of the sequence CAGTGACTGTCAGGTCGATCATATCCATG (Origene) into the pGFP-V-RS vector. The pGFP-V-RS-shScramble plasmid, whose inserted sequence does not match any known human cDNA, was used as control. The mammalian expression plasmid for RFP-ALG-2 was constructed by insertion of the ALG-2 cDNA into the pcDNA3.1-RFP vector. Human ALG-2 siRNAs were described previously [20], and mALG-2 siRNA (5′-GGAUCAGGAUGGCUGGAUU-3′) was synthesized by Ribobio. Plasmids were transfected to cells using the Lipofectamine 3000 reagent (Invitrogen), and siRNAs were transfected using Lipofectamine RNAiMAX (Invitrogen).

### Cell culture

MDA-MB-231 and BT549 human breast cancer cells, MCF-10A immortalized human breast epithelial cells, and HEK293T human embryonic kidney epithelial cells were purchased from the American Type Culture Collection. The 4T1-luc luciferase-expressing mouse breast cancer cells were described previously [21]. To generate stable ALG-2-knockdown MDA-MB-231 cells (referred to as MDA-MB-231/shALG-2), the pGFP-V-RS-shALG-2 retroviral plasmid and the packaging plasmids pVSVG and pMLV-Gag-Pol were transfected into HEK293T cells for 48 hours. The virus-containing supernatant was harvested, filtered, and concentrated by ultracentrifugation and transduced into MDA-MB-231 cells with polybrene (Sigma-Aldrich). Stable ALG-2-knockdown cells were then selected with puromycin (Invitrogen) as described previously [22]. All cells were cultured as described by the American Type Culture Collection supplemented with 10% fetal bovine serum at 37°C in a humidified atmosphere with 5% CO_2_.

### Human tissue samples and immunohistochemistry

Human breast cancer tissues and adjacent tissues were obtained from patients who underwent surgical resection at Shanxian Dongda Hospital, Shandong, China. The use of human tissues was approved by our Institutional Ethics Committee. Paraffin-embedded breast tissue sections were cut, dewaxed, and rehydrated with xylene and graded alcohols. After antigen retrieval and inactivation of endogenous peroxidase, sections were blocked with goat serum and incubated with primary antibodies. Subsequently the sections were incubated with biotinylated secondary antibody and streptavidin-biotin-peroxidase, using daminobenzidine as the chromogen substrate, and finally haematoxylin counterstaining was performed. The expression of ALG-2 was graded according to the staining intensity (0 = negative; 1 = low; 2 = medium; 3 = high) and the percentage of stained cells (0 = 0% stained; 1 = 1%–25% stained; 2 = 26%–50% stained; 3 = 51%–100% stained) as described previously [23, 24].

### Immunoblotting

Proteins were resolved by SDS/PAGE and transferred onto polyvinylidene difluoride membranes (Millipore). The membranes were blocked in Tris-buffered saline containing 0.1% Tween 20 and 5% fat-free milk for 2 hours at room temperature, and incubated first with primary antibodies and then with horseradish peroxidase-conjugated secondary antibodies. Specific proteins were visualized with enhanced chemiluminescence detection reagent according to the manufacturer's instructions (Pierce).

### Tumor growth and metastasis in mice

All procedures for mouse care and operation were carried out according to the guidelines of experimental animals. The use of mice was approved by our Institutional Animal Care Committee. To examine tumor growth, cells were injected subcutaneously into the right flank of female athymic nude mice, and tumor volumes were measured with a vernier caliper and calculated as described previously [25]. After the mice were sacrificed, tumors were isolated, photographed, weighed and sectioned for immunohistochemistry, hematoxylin and eosin (HE) staining, and immunofluorescence staining. To analyze tumor metastasis, 4T1-luc cells were injected into the lateral tail vein of female BALB/c mice as described previously [21]. Luciferin (150 mg/kg) was injected intraperitoneally 15 minutes before imaging. Metastatic cells were monitored with an IVIS Imaging System (Xenogen). Mice were sacrificed 6 days later and tumors were harvested for bioluminescent imaging or sectioned for HE staining.

### Cell proliferation and colony formation

For cell proliferation, 5 × 10^3^ cells were seeded in 96-well plates, and the density of cells was determined by SRB and MTT assays after various days. For SRB assays, cells were fixed with 50% trichloroacetic acid and stained with 0.4% SRB dissolved in 1% acetic acid. The cells were then washed with 1% acetic acid to remove unbound dye. The protein-bound dye was extracted with 10 mM Tris base to determine the optical density at 490 nm wavelength. The MTT assays were performed by using the Vybrant MTT cell proliferation assay kit following the manufacturer's instructions (V13154, Thermo Fisher). For colony formation, 500 cells were seeded in six-well plates and cultured for 2 weeks. The colonies were fixed with methanol and stained with 0.1% crystal violet. The number of colonies in each well was then counted.

### Flow cytometric analysis

Cells were stained with annexin V-FITC and propidium iodide using the Vybrant apoptosis assay kit (Invitrogen). In brief, cells were collected by trypsinization and washed with ice-cold phosphate-buffered saline (PBS). Cells were resuspended and incubated with annexin V-FITC and propidium iodide at room temperature for 5 minutes in the dark. Cells were then analyzed by a Coulter Elite flow cytometer (BD) as described [26].

### Fluorescence microscopy

Cells grown on glass coverslips were fixed with 4% paraformaldehyde/ PBS for 30 minutes, followed by permeabilization in 0.5% Triton X-100/PBS for 20 minutes, or fixed with methanol at −20°C. Paraffin-embedded breast tissue sections were cut, dewaxed, and rehydrated with xylene and graded alcohols and then underwent antigen retrieval and inactivation of endogenous peroxidase. Cells or tissues were blocked with 2% bovine serine albumin in PBS and incubated with primary antibodies and then FITC- or rhodamine-conjugated secondary antibodies, followed by staining with DAPI as described [27]. For visualization of microfilaments, cells were stained with rhodamine-conjugated phalloidin for 30 minutes. Coverslips were then mounted and examined with an Axio Observer A1 fluorescence microscope (Carl Zeiss) as described previously [28].

### Cell migration and invasion

For wound healing experiments, cells cultured in 24-well plates were scratched with a 10 μL pipette tip to generate the wound. Cells were then washed twice with PBS to remove the cell debris. Photographs of the wound were taken to determine the extent of wound closure. For transwell experiments, cells were seeded to the upper chamber of transwell filters (BD) in serum-free media in triplicate in a 24-well tissue culture plate. The lower chambers were filled with media supplemented with 2% fetal bovine serum. Cells on the inside of the inserts were removed with a cotton swab, and invaded cells underside were fixed with 4% paraformaldehyde, stained with the crystal violet solution, and imaged. For cell tracking assays, cells were cultured in a 35-mm dish overnight. The migration of the cells was recorded at an interval of 5 minutes for 12 hours with a phase contrast microscope as described previously [29, 30]. Tracks and migration velocities were determined with the Image J software (NIH).

### Microtubule depolymerization and regrowth

Microtubule depolymerization and regrowth experiments were performed as described previously [31]. Briefly, cells were incubated on ice for different time to depolymerize microtubules. After all microtubules were depolymerized, cells were placed back at 37°C for different time. Cells were then stained with anti-α-tubulin antibody and DAPI and examined with a fluorescence microscope. Microtubule intensity was measured with the Image J software.

### Statistics

Analysis of statistical significance was performed by the Student's *t*-test for comparison between two groups and by the ANOVA test for multiple comparisons. Correlation coefficient was calculated by the Spearman's rank correlation test.
